# Superstition in arthroplasty: does a suspicious size or surgery date have a higher revision rate? A Dutch arthroplasty register study

**DOI:** 10.2340/17453674.2025.44594

**Published:** 2025-08-19

**Authors:** Jeroen C VAN EGMOND, Jantsje H PASMA, Liza N VAN STEENBERGEN, Olav P VAN DER JAGT

**Affiliations:** 1 Department of Orthopedic Surgery, Bravis Ziekenhuis, Roosendaal; 2 Department of Orthopedic Surgery, Reinier Haga Orthopedic Center, Zoetermeer; 3 Dutch Arthroplasty Register (Landelijke Registratie Orthopedische Interventies/LROI),’s-Hertogenbosch; 4 Department of Orthopedic Surgery, Elisabeth-TweeSteden ziekenhuis, Tilburg, The Netherlands

## Abstract

**Background and purpose:**

There are still strong beliefs in medicine concerning things that bring “bad luck.” It is unclear whether a suspicious component size or surgery date is related to “bad luck” in orthopedic surgery. We aimed to examine: (i) if a potentially unlucky femoral stem size 13 in total hip arthroplasty (THA), and (ii) if a possible unlucky surgery date, Friday 13th, in THA and total knee arthroplasty (TKA) have a higher revision rate.

**Methods:**

We analyzed 611,050 THAs and TKAs, performed in the past 13 years using Dutch Arthroplasty Register data. The revision rate of uncemented femoral stem size 13 (Corail and Taperloc) in THA was compared with all other stem sizes of the same type. Furthermore, the revision rate of THA and TKA implanted on Friday 13th was compared with all other days and with other Fridays. Both were performed using competing risk analyses with death as competing risk and cause-specific multivariable Cox proportional hazard regression analyses.

**Results:**

The use of an uncemented Corail or Taperloc femoral stem size 13 in THA was associated with a lower revision rate (3.0%, 95% confidence interval [CI] 2.3–4.0) compared with the revision rate of other femoral stem sizes (3.5%, CI 3.3–3.8) (hazard ratio [HR] 0.76, CI 0.65–0.87). TKA procedures on Friday 13th were not associated with increased revision rate (5.2%, CI 4.1–6.7) compared with procedures on other days (6.0%, CI 5.9–6.2) or on other Fridays (5.8%, CI 5.4–6.2) (HR 1.03, CI 0.80–1.32 and HR 1.01, CI 0.79–1.30, respectively). For THA, procedures on Friday 13th were associated with a higher revision rate (5.1%, CI 3.9–6.6) compared with procedures on other days (4.6%, CI 4.5-4.8) (HR 1.32, CI 1.04–1.67) but not compared with procedures on other Fridays (4.8%, CI 4.4–5.1) (HR 1.24, CI 0.97–1.58).

**Conclusion:**

Based on national arthroplasty registry data, femoral stem size 13 in THA was associated with a lower revision rate. TKA procedures on Friday 13th were not associated with increased revision rate; however, in THA there seems to be an increased risk of revision in THA procedures performed on Friday 13th compared with other days, but not when compared with other Fridays.

Doctors tend to have a fierce commitment to the rational. However, superstition in medicine has been there for a long time and many patients are influenced by it [1]. Moreover, myths and traditions are still present in hip and knee arthroplasty and influence treatment as described in the article by Husted et al. (2014) [2]. Superstition is a belief that is not based on scientific knowledge [1]. There are still strong beliefs in medicine regarding things that bring “bad luck,” for instance, a full moon, saying the word “quiet” during an on-call period, or Friday 13th [3-6].

Triskaidekaphobia, the fear of number 13, is very often seen in Western Europe and North America, but other areas and countries can have other unlucky numbers. For instance, in Italy number 17 is avoided, because since Roman times this number is related to death and bad fortune. Furthermore, in Japan the numbers 4 and 9 are unlucky numbers, because their pronunciation looks like respectively death and suffering. And another difference in superstition can be found for Friday 13ths. In Latin America and Spain, it is not the Fridays that are related to bad luck but Tuesday the 13ths. Other known and commonly investigated factors are for example lunar phases or zodiac signs [3,7,8]. As well as unlucky numbers, lucky numbers also exist. Lucky numbers seem very individual, but in general the numbers 10 (the “perfect” number) and number 8 (infinity) are often mentioned.

Almost 30 years ago, the study by Scanlon et al. (1993) showed that Friday 13th is unlucky for some and staying home is recommended, as there is a higher risk of transport accidents [9]. Still, the number 13 is avoided in numbering airplane seats, surgery rooms, hospital rooms, and floors [9]. However, recently Nardelli et al. (2023) found no relation between lunar phase and surgery on Friday 13th with revision-free survival of total knee arthroplasty (TKA) [8]. Moreover, no influence of lunar phase, zodiac signs, or Friday 13th was found in surgical blood loss and emergency frequency [10,11]. Also, lunar phase has no effect on perioperative complications in total hip arthroplasty (THA) [7]. Recently Ranganathan et al. (2024) found similar outcomes of surgical procedures on Friday 13th compared with flanking Fridays [12].

It is unclear whether a suspicious component size is related to “bad luck.” Therefore, the question remains as to whether “triskaidekaphobia” is motivated and if this also applies to arthroplasty. We questioned (i) whether an uncemented femoral stem size 13 (Taperloc or Corail) in THA has a higher risk of revision, and (ii) does primary THA or TKA performed on Friday 13th have increased risks of revision?

## Methods

### Study design and data source

Data was retrieved from the Dutch Arthroplasty Register (Landelijke Registratie Orthopedische Interventies: LROI). The LROI is a nationwide population-based register, initiated by the Dutch Orthopaedic Association. The register contains information on joint arthroplasties in the Netherlands since 2007, with over 97% completeness of all primary and revision hip and knee arthroplasties performed in the Netherlands [13]. All data obtained from the LROI database were prospectively collected. The study is reported according to STROBE reporting guidelines.

### Study design and population

All patients who underwent primary TKA and THA for osteoarthritis implanted in the Netherlands during the past 13 years, namely between January 1, 2010 and January 1, 2023 and registered in the LROI were included for analysis. Procedures were excluded when reason for arthroplasty was other than osteoarthritis.

**Figure 1:**
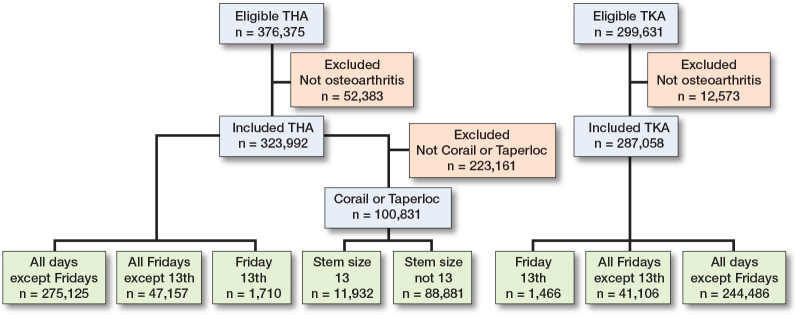
Flowchart of numbers included in the analysis of the association of femoral size 13 with revision and the association of Friday 13th with revision in total knee arthroplasty and total hip arthroplasty.

To examine the association of femoral stem size 13 and other femoral stem sizes with the revision rate, all primary uncemented THAs with Corail (DePuy Synthes, Leeds, England) or Taperloc (Zimmer Biomet, Warsaw, IN, United States) hip stem were selected, because only these manufacturers use femoral stem size 13 (Figure 1).

To investigate the association of Friday 13th, other Fridays, and other days except 13th with the revision rate, all manufacturers for THA and TKA were included for analysis. A total of 611,050 arthroplasties were included (THA n = 323,992, TKA n = 287,058). Procedures on Fridays except 13th, and on Friday 13th were selected (Figure 1).

### Outcome

A revision was defined as any exchange (placement, replacement, or removal) of 1 or more primary implanted components.

### Statistics

Survival analysis was performed of femoral stem size 13 in THA compared with all other stem sizes. Moreover, survival analyses were used to compare THAs and TKAs performed on Friday 13th with (i) all other days (including other Fridays, Saturday, and Sunday) and (ii) other Fridays. Analyses were performed in accordance with guidelines for statistical analysis of arthroplasty register data [14,15]. Case characteristics and surgical details were stratified for hip stem size and surgery date.

Survival time was defined as the time from primary THA or TKA to first (any) revision arthroplasty for any reason, death of the patient, or January 1, 2023. The follow-up time was calculated by the reverse Kaplan–Meier estimated potential follow-up as described by Schemper and Smith [16]. Competing risk analyses were used to calculate the 13-year crude cumulative incidence of revision for any reason with death as competing risk. Cause-specific multivariable Cox proportional hazard regression analyses were used to compare revision rates between procedures with a Corail or Taperloc femoral stem size 13 and procedures with Corail or Taperloc stems of other sizes adjusted for age at surgery and body mass index (BMI). Furthermore, cause-specific multivariable Cox proportional hazard regression analyses were used to compare revision rates between (i) procedures on Friday 13th and procedures on another day adjusted for age at surgery and BMI, and (ii) procedures on Friday 13th and procedures on other Fridays. In case of an expected weekend effect (i.e., the effect of surgery on the day before the weekend [Friday] on the revision rate), cause-specific multivariable Cox proportional hazard regression analyses were performed to compare revision rates between procedures on Friday and procedures on another day (not on Friday) adjusted for age at surgery and BMI. All analyses were stratified for THA and TKA. Age at surgery and BMI were included as covariates due to their expected association with outcome (revision) and exposure (choice of femoral stem size or day of surgery). The proportional hazards assumptions were checked for all included covariates by inspecting log-minus-log curves. In case the covariate did not fulfil the proportional hazards assumption, the covariate was included as time-varying variable.

For all statistical analyses IBM SPSS statistics version 28 (IBM Corp, Armonk, NY, USA) was used. P values of 0.05 or lower were considered to be statistically significant.

### Ethics, data sharing, funding, and disclosures

All data was registered confidentially with patient consent as part of routine clinical care and in accordance with Dutch and EU data protection rules. The present study placed no additional burden on the patient. Therefore, no ethical approval was necessary according to the Dutch Medical Research Involving Human Subjects Act (WMO). The study was approved by the board and scientific advisory committee of the Dutch Arthroplasty Register. All data was collected anonymously and handled in line with the Declaration of Helsinki (version 64, October 2013). Sharing of data is not permitted by the LROI due to privacy regulations. The authors received no financial or material support for the research, authorship, and/or publication of this article. The authors report no conflict of interests. Complete disclosure of interest forms according to ICMJE are available on the article page, doi: 10.2340/17453674.2025.44594

## Results

### Femoral stem size 13

**Figure 2:**
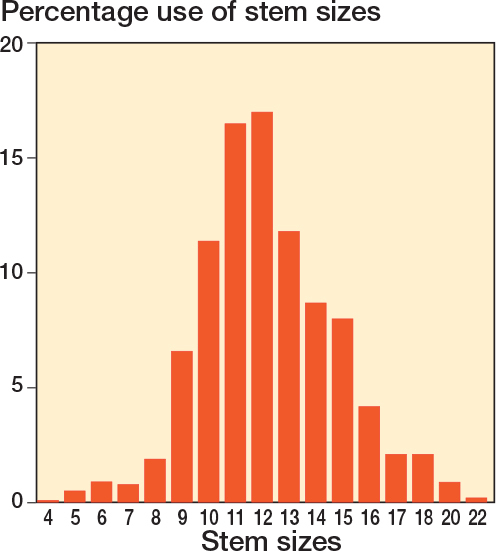
Distribution of implanted Corail and Taperloc stem size.

In total, 100,831 THA were performed with a Corail or Taperloc stem of the 323,992 THAs (Figure 1). Femoral stem size 13 was used in 11,932 (12%) procedures. The patient characteristics are comparable between groups, with an overall mean (SD) age of 79.7 (9.7) years and 110,250 (34%) men (Table 1). Corail and Taperloc femoral stem sizes seem to be normally distributed. Sizes 19 and 21 do not exist (Figure 2).

**Table 1:** Characteristics of all cases with primary total hip arthroplasty surgery with a Corail or Taperloc stem between 2010 and 2022 in the Netherlands stratified by femoral stem size and stratified by performance on all days except Friday, all Fridays except Friday 13th, and Friday 13th. Values are count (%) unless otherwise specified

Item	All THA (n = 323,992)	Stratification by stem and stem size			Stratification by day of surgery		
		All Corail and Taperloc (n = 100,813)	Corail or Taperloc, not size 13 (n = 88,881)	Corail or Taperloc, size 13 (n = 11,932)	THA on all days except Friday (n = 275,125)	THA on Friday except Friday 13th (n = 47,157)	THA on Friday 13th (n = 1,710)
Age at surgery (SD)	69.7 (9.7)	67.8 (9.3)	67.6 (9.3)	68.5 (9.0)	69.6 (9.7)	69.9 (9.9)	70.4 (9.6)
Male sex	110,250 (34)	37,705 (37)	32,544 (37)	5,161 (43)	93,686 (34)	15,953 (34)	611 (36)
BMI (SD)	27.4 (4.5)	27.3 (4.4)	27.4 (4.4)	27.2 (4.3)	27.4 (4.5)	27.5 (4.6)	27.7 (4.6)
ASA class							
I	58,528 (18)	21,373 (21)	18,937 (21)	2,436 (20)	50,001 (18)	8,260 (18)	267 (16)
II	207,948 (64)	64,256 (64)	56,720 (64)	7,536 (63)	177,356 (65)	29,489 (62)	1,103 (65)
III–IV	56,240 (17)	14,861 (15)	12,931 (15)	1,930 (16)	46,662 (17)	9,244 (20)	334 (19)
Smoking	23,470 (7.2)	8,626 (8.6)	7,549 (8.5)	1,077 (9.0)	9,888 (7.2)	3,451 (7.3)	131 (7.7)
Previous surgery	5,786 (1.8)	1,296 (1.3)	1,153 (1.3)	143 (1.2)	4,837 (1.8)	915 (1.9)	34 (2.0)

Numbers do not add up to 100% due to missing data.

BMI = body mass index; ASA = American Society of Anesthesiologists.

**Figure 3:**
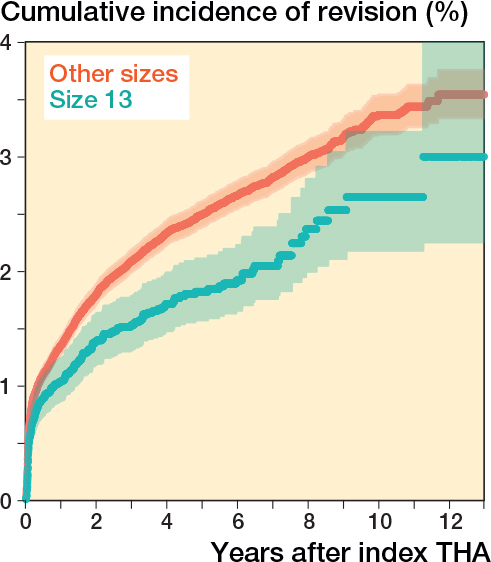
Crude cumulative incidence of revision, according to stem size in total hip arthroplasty with osteoarthritis in the Netherlands in the period 2010–2022.

13-year cumulative incidence function for both the femoral stem size 13 and all other stem sizes in THA showed a lower cumulative incidence function in femoral stem size 13 compared with other stem sizes (Figure 3). After adjustment for age at surgery and BMI, THA procedures with a femoral stem size 13 showed a lower revision rate compared with other stem sizes (hazard ratio [HR] 0.76, 95% confidence interval [CI] 0.65–0.87) (Table 2).

**Table 2:** Number of events and survival for revision in Corail and Taperloc stems between 2010 and 2022 in the Netherlands stratified by size

Item	Corail or Taperloc		Adjusted HR (CI) **^a^**
	not size 13	size 13	(n = 99,831)
Follow-up (CI) **^b^**	4.4 (4.4–4.4)	4.0 (3.9–4.0)	
Revised, n (%)	2,100 (2.4)	203 (1.7)	
CIF **^c^**, % (CI)	3.5 (3.3–3.8)	3.0 (2.3–4.0)	0.76 (0.65–0.87)

a Hazard ratio adjusted for BMI and age at surgery.

b Median follow-up in years

c CIF = Cumulative incidence function of revision at 13 years’ FU.

CI = 95% confidence interval; FU = follow-up.

### Friday 13th

Between 2010 and 2022 there were a total of 22 Friday 13ths. Of the 297,058 TKAs and 323,991 THAs, 1,466 TKAs and 1,710 THAs were performed on Friday 13th (Tables 1 and 3 and Figure 1). On other Fridays, 41,106 TKAs and 47,157 THAs were performed.

**Table 3:** Characteristics of all cases with primary total knee arthroplasty surgery between 2010 and 2022 in the Netherlands stratified by performance on all other days except Friday, all other Fridays except Friday 13th, and Friday 13th. Values are count (%) unless otherwise specified

Item	All TKA (n = 287,058)	TKA on all days except Friday (n = 244,486)	TKA on Friday except Friday13th (n = 41,106)	TKA on Friday 13th (n =1,466)
Age at surgery (SD)	68.9 (9.0)	68.8 (9.0)	69.2 (9.1)	69.6 (9.0)
Male sex	102,906 (36)	88,020 (36)	14,377 (35)	509 (35)
BMI (SD)	29.7 (5.0)	29.6 (5.0)	29.9 (5.1)	29.8 (5.2)
ASA				
I	40,075 (14)	34,734 (14)	5,174 (13)	167 (11)
II	191,377 (67)	163,616 (67)	26,793 (65)	968 (66)
III–IV	53,777 (19)	44,558 (18)	8,895 (22)	324 (22)
Smoking	17,001 (5.9)	14,600 (6.0)	2,309 (5.6)	92 (6.3)
Previous surgery	78,776 (27)	68,028 (28)	10,394 (25)	354 (24)

See footnote to Table 1.

**Figure 4:**
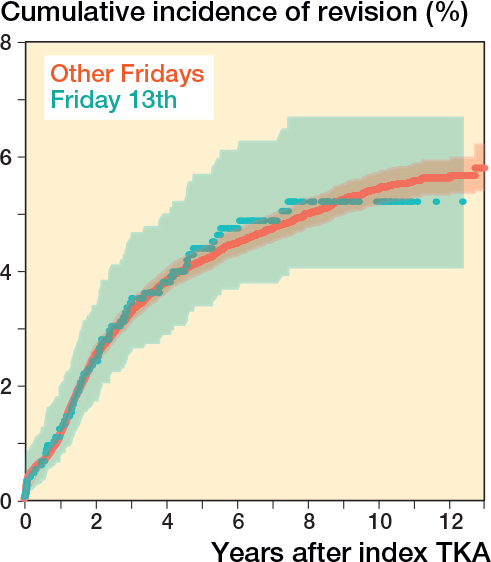
Crude cumulative incidence of revision, according to Friday and Friday 13th for total knee arthroplasty in the Netherlands in the period 2010–2022.

For TKA, procedures on Friday 13th have the same revision rate compared with procedures on another day after adjustment for age at surgery and BMI (HR 1.03, CI 0.80–1.32) (Table 4). The 13-year cumulative incidence function of revision for TKAs performed on Friday 13th and TKAs performed on other Fridays showed comparable cumulative incidence functions (Figure 4). Moreover, TKA procedures on Friday 13th have the same revision rate compared with procedures on another Friday (HR 1.01, CI 0.79–1.30) (Table 5).

**Table 4:** Number of events and survival for revision in total knee arthroplasty between 2010 and 2022 in the Netherlands stratified by Friday 13th and other days (including other Fridays)

Item	On	On	Adjusted HR (CI) **^a^**
	other days	Friday 13th	(n = 283,924)
Follow-up (CI) **^b^**	5.7 (5.7–5.7)	6.0 (5.8–6.2)	
Revised, n (%)	11,824 (4.1)	63 (4.3)	
CIF **^c^**, % (CI)	6.0 (5.9–6.2)	5.2 (4.1–6.7)	1.03 (0.80–1.32)

a, b, c See Table 2.

**Table 5:** Number of events and survival for revision in total knee arthroplasty between 2010 and 2022 in the Netherlands stratified by Friday 13th and other Fridays

Item	On	On	Crude HR (CI)
	other Fridays	Friday 13th	(n = 42,572)
Follow-up (CI) **^b^**	5.6 (5.5–5.7)	6.0 (5.8–6.2)	
Revised, n (%)	1,681 (4.1)	63 (4.3)	
CIF **^c^**, % (CI)	5.8 (5.4–6.2)	5.2 (4.1–6.7)	1.01 (0.79–1.30)

b, c See Table 2.

**Figure 5:**
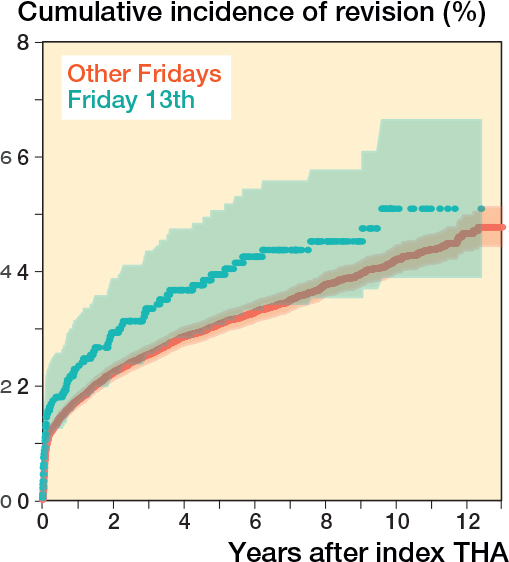
Crude cumulative incidence of revision, in relation to Friday and Friday 13th in total hip arthroplasty in the Netherlands in the period 2010–2022.

For THA, procedures on Friday 13th have an increased revision rate compared with procedures on other days (HR 1.32, CI 1.04–1.67) (Table 6). The 13-year cumulative incidence function of THAs performed on Friday 13th compared with THAs performed on other Fridays are comparable (Figure 5). When comparing procedures on Friday 13th with only procedures on other Fridays, no difference was shown (HR of 1.24 (CI 0.97–1.58)) (Table 7).

**Table 6:** Number of events and survival for revision in total hip arthroplasty between 2010 and 2022 in the Netherlands stratified by Friday 13th and other days (including other Fridays)

Item	On	On	Adjusted HR (CI) **^a^**
	other days	Friday 13th	(n = 320,716)
Follow-up (CI) **^b^**	5.3 (5.3–5.3)	5.2 (5.0–5.4)	
Revised, n (%)	9,837 (3.1)	69 (4.0)	
CIF **^c^**, % (CI)	4.6 (4.5–4.8)	5.1 (3.9–6.6)	1.32 (1.04–1.67)

a, b, c See Table 2.

**Table 7:** Number of events and survival for revision in total hip arthroplasty between 2010 and 2022 in the Netherlands stratified by Friday 13th and other Fridays

Item	On	On	Crude HR (CI)
	other Fridays	Friday 13th	(n = 48,867)
Follow-up (CI) **^b^**	5.1 (5.1–5.2)	5.2 (5.0–5.4)	
Revised, n (%)	1,506 (3.2)	69 (4.0)	
CIF **^c^**, % (CI)	4.8 (4.4–5.1)	5.1 (3.9–6.6)	1.24 (0.97–1.58)

b, c See Table 2.

When comparing all THA procedures performed on Fridays with other weekdays a minor increased revision rate was found (HR 1.08, CI 1.02–1.14]) (Table 8).

**Table 8:** Number of events and survival for revision in total hip arthroplasty between 2010 and 2022 in the Netherlands stratified by Fridays (including Friday 13th) and other days

Item	On	On	Adjusted HR (CI) **^a^**
	other days	Fridays	(n = 320,716)
Follow-up (CI) **^b^**	5.3 (5.3–5.4)	5.2 (5.1–5.2)	
Revised, n, (%)	8,331 (3.0)	1,575 (3.2)	
CIF **^c,^** % (CI)	4.6 (4.5–4.8)	4.8 (4.5–5.1)	1.08 (1.02–1.14)

a, b, c See Table 2.

## Discussion

The objective of our study was to analyze whether orthopedic surgeons and/or patients should worry about triskaidekaphobia (fear and/or avoidance of number 13). We found no reasons to avoid femoral stem size 13 in THA. In fact, femoral stem size 13 can be placed with high confidence, because revision rates are lower when compared with all other sizes. Furthermore, our analysis reveals that Friday 13th is just another Friday for TKA. However, the hazard of revision was increased for THA implanted on Fridays 13th compared with other days, although THA implanted on Friday 13th showed no increased hazard of revision when compared with other Fridays. Finally, when THAs implanted on all Fridays were compared with all other days a small increased hazard of revision was found, which might suggest a weekend effect.

Like everyone, surgeons can suffer from superstition and because the goal in THA is to give a well-functioning implant for a very long time, one might be tempted to avoid femoral stem size number 13 on the grounds that this could bring bad luck. We found a natural distribution of stem sizes and size 13 seems not to be avoided. But for those surgeons who in their subconscious still question the reliability of size 13 we can now conclude that this fear is unfounded. In fact, size 13 seems to be protective for revisions for any reason when compared with all other sizes.

Since we analyzed only revisions for all reasons, we cannot conclude that this finding is related to stem size only. Hence, revisions can also be related to cup failure, dislocation, infection, etc. Furthermore, we compared size 13 with all other sizes, which might indicate that there might be a higher risk for revisions with less regular sizes, but further analysis is beyond the scope of this paper.

The absent of an association of Friday 13th with the revision rate of TKA is in accordance with the recently published study by Nardelli et al., who also did not find a relationship between surgery on Friday 13th and revision in TKA [8]. Despite their large, included group of 5,932 procedures, only 21 TKA patients had surgery on Friday 13th, which might underpower their subgroup analysis. In the current study we used data from a national arthroplasty register to include a large number of patients. In contrast to the study of Nardelli et al. no preoperative patient-reported outcome measures (PROMs) were evaluated.

We presumed that surgeries performed on Fridays are comparable to all other surgery days and that difficult patients are not scheduled on other days because of the weekend. However, for THA we found a lower, but not significant, association when comparing Friday 13th with other Fridays instead of all other days. Although the difference in hazard of revision for THA between Fridays and other days is very small, we do see a statistically significant difference which is not observed in TKA.

### Strength

The strength of this study was the large number of procedures included for analysis. Because a national arthroplasty register was used, we presume that our findings are robust and generalizable.

### Limitations

There is a lack of knowledge of complications other than revision. For example, a DAIR (Debridement, Antibiotics and Implant Retention) procedure without change of any of the components is not registered in the arthroplasty register. As no information is available on comorbidity and socioeconomic background it is not possible to correct for these confounders that may influence outcome [17]. However, there is no reason to believe that this should be different between groups.

### Conclusion

Based on 13-year prospective collected data from a national arthroplasty registry we can conclude that there is no basis to avoid 13 in surgery theatre. Orthopedic surgeons should not hesitate to use an uncemented femoral stem size 13. Although there was an increased risk of revision in THA procedures on Friday 13th compared with other days, but not when compared with other Fridays, surgery on Friday 13th can be performed with the same confidence as on other Fridays.

We are convinced that for those orthopedic surgeons who might have (subconscious) triskaidekaphobia, but also for patients with triskaidekaphobia this data is helpful to convince them to face number 13 like any other number and that surgical decisions are made by reason and not by phobias.

## Notes

JE designed the study, supported data analysis, and he wrote and critically reviewed the manuscript. JP supported data collection, data analysis, and critically reviewed the manuscript. LS provided data from the Dutch Arthroplasty Register (LROI), supported data analysis, and critically reviewed the manuscript. OJ designed the study, supported data analysis, and critically reviewed the manuscript. All authors approved the final version of the manuscript.

The authors would like to thank the LROI for use of the data.

Handling co-editors: Keijo Mäkelä and Robin Christensen

*Acta* thanks Eduardo García-Rey and Ove Nord Furnes for help with peer review of this manuscript.
